# Stress-related experiences and intentions to quit studies among female married postgraduate distance education students in Ghana

**DOI:** 10.1186/s40359-024-01839-x

**Published:** 2024-06-14

**Authors:** Eugene Adu Henaku, Francis Sambah, Frank Quansah, Edmond Kwesi Agormedah, Medina Srem-Sai, John Elvis Hagan, Francis Ankomah, Vera Rosemary Ankomah-Sey, Thomas Schack

**Affiliations:** 1 Department of Family Life Management Education, University of Education, P. O. Box 25, Winneba, Ghana; 2 College of Public Health, Medical and Veterinary Sciences, James Cook University, Townsville, QLD 4811 Australia; 3 Department of Educational Foundations, University of Education, P. O. Box 25, Winneba, Ghana; 4 Department of Business & Social Sciences Education, University of Cape Coast, PMB Cape Coast, Cape Coast, Ghana; 5 Department of Health, Physical Education, Recreation and Sports, University of Education, P. O. Box 25, Winneba, Ghana; 6 Department of Health, Physical Education and Recreation, University of Cape Coast, PMB Cape Coast, Cape Coast, Ghana; 7 Neurocognition and Action-Biomechanics-Research Group, Faculty of Psychology and Sports Science, Bielefeld University, Postfach 10 01 31, 33501 Bielefeld, Germany; 8 Department of Education and Psychology, University of Cape Coast, PMB Cape Coast, Cape Coast, Ghana; 9 Department of Educational Studies, Patton College of Education, Ohio University, 45701, Athens, OH, USA; 10 College of Distance Education, University of Cape Coast, PMB Cape Coast, Cape Coast, Ghana

**Keywords:** Stressors, Married women, Distance education, Gender, Postgraduate studies

## Abstract

**Background:**

Although postgraduate studies have been shown to be associated with stressful experiences, students reading programmes through the distance and e-learning mode experience greater levels of stress due to several reasons. These stressful encounters might be heightened in female married postgraduates on distance education programmes due to other family-work-related engagements. This study investigated the stress-related experiences and intentions to quit studies among female married students on a distance education programme in Ghana.

**Methods:**

Using a sequential explanatory mixed-methods design, 164 married postgraduate distance education students were sampled to participate by responding to a questionnaire. Follow-up interviews were conducted with 10 participants to offer insight into the quantitative findings. Quantitative data were analysed using descriptive statistics, including frequency and percentages, while the qualitative data were thematically analysed.

**Results:**

Stress was prevalent among the female married distance education students, with the majority having intentions of quitting their studies. The stressors identified ranged from personal (i.e., work and family demands) to institutional ones (i.e., academic load, unresolved complaints and high financial demands from the programme).

**Conclusions:**

Key findings suggest that female married postgraduate distance education students perform multiple roles as full-time employees with family and academic demands that can negatively impact their health and academic work. Implications and recommendations of the findings are discussed.

## Introduction

The popularity of distance learning has grown recently. Parallel to this growth, advocates for education reform, including educators, policymakers and researchers, are increasingly calling for an action in response to the psychological consequences (stress) and intention to discontinue studies (student attrition or dropout rate) in the distance education space. Although distance education students enjoy a more flexible learning environment, they nonetheless experience stress, which influences their psychological well-being and academic progress [1, 2]. Stress in this study reflects the conceptualization provided by Lazarus and Folkman [3] as “a particular relationship between the person and the environment that is judged by the person as burdensome or exceeding his or her capacity or resources to cope with the situation at hand” (p. 19). Thus, the disproportion between demands and resources is characterized as stress [4]. More specifically, we describe “educational or academic stress as the sensation of being overburdened by school workloads or demands”.

Studies have reported that students enrolled in distance education worldwide have been shown to experience high levels of stress [5–7]. Given that males and females react to stress differently [8], scholars have found that female distance learners exhibit higher levels of academic stress than their male counterparts [9–11]. Particularly, married women enrolled in distance education programmes are also more likely than men to experience and exhibit symptoms of depression, anxiety, discomfort, and stress [12, 13]. These stressors are linked to several internal (e.g., personal goals, expectations, standards) [2, 14] and external stressors (e.g., excessive academic workload and assignments, time constraints, financial issues, attending to family-related responsibilities, and other social issues) [14–23, 14]. Although educational stress can be beneficial because it challenges students and motivates students to succeed [24], it can also have negative effects on student learning [25, 26], judgment and adaptive function [23] and lead to examination malpractices, and engaging in inappropriate school behaviours [26–28]. Stress can cause unhealthy behaviours [29, 30] and is linked to students’ intentions to drop out of school [31, 32].

In higher education, the intention of distance education students to quit or drop out is on the rise [33–36]. In this current investigation, students’ intention to quit a study is defined as “the perceived or subjective probability that a student will drop out of the programme in which they have enrolled and the frequency of having that thought”. According to earlier research reports [37, 38], dropping out of school has a negative impact on students’ self-esteem, psychological, emotional, and social well-being. This outcome has a negative impact on learning, unemployment, and lower standard of living. Extant researchers have found that distance education students’ intention to quit studies or drop out is related to several factors, including psychological consequences such as stress and burnout symptoms [39–44]. Swani et al. [45] established that stress reduces students’ satisfaction and intention to stay on their programme. In Peru, it was found that most distance education students withdrew from their studies due to mental health-related reasons [34, 46, 47]. In Brazil, De Souza et al. [48] showed that distance education students’ dropout intention was influenced by negative affectivity (i.e., symptoms of depression, anxiety, and stress). The effect of academic stress on the intention to quit studying was attributed to several reasons, such as conflicts between study, work/employment, and family commitments [49, 50]. The online and distance education literature points to the challenges of balancing work, study, and family responsibilities as the reasons for dropping out of a university [49–52].

Due to inadequate or deteriorating facilities, universities in developing economies, such as Ghana, have had to deal with the unpleasant responsibility of rejecting qualified applicants [53]. As a result, various universities use distance education as one of the most effective teaching methods to expand access to tertiary education to meet the growing demands of professional education [54, 55]. Most educators believe that traditional on-campus education and training can be improved by distance learning. Distance education has become a way for existing and new educational establishments to admit working-class students ever since it was introduced [56]. In Ghana, for example, the majority of distance learners are adults who are married, parents, or employed (working), placing a huge demand on their learning time. Thus, these learners are likely to frequently face psychological, socio-religious, and financial/economic difficulties, as a result of creating a balance between work, school, and social lives. In fact, previous studies in Ghana have confirmed that student mothers in distance education face challenges such as the inability to regularly attend face-to-face lectures due to exhaustion, poor health of their children, and the lack of lactation rooms for breastfeeding [57]. Other findings have shown that married students were more stressed than unmarried students due to institutional, instructional, social, psychological, financial and family/marriage problems [54, 58, 59]. Obviously, these conflicts put a lot of pressure on these learners and negatively impact their academic performance [60–62].

During face-to-face interactions with female married distance students in some of the distance education campuses of one of the public universities in Ghana, they complained of headaches, lack of sleep, and fatigue due to poor educational services received from the university. Accordingly, frustrated distance learners who are unable to cope with the stress may delay on or withdraw from the academic programme, thereby squandering their investment and jeopardizing their educational goals [63, 64]. Studies on distance education in Ghana have focused mainly on stress, struggles and coping strategies [54, 57, 62], challenges and coping strategies [59], satisfaction and choice of distance programme [53, 60], students’ learning with information communication technology [65], and student mode of learning [66]. Yet issues of how these stressors manisfest and intentions to quit the programme have not been extensively investigated, especially with a unique population like female married women. Given this premise, this study examined female married postgraduate distance education students’ stress-related experiences and intention to quit studies. The current inquiry (1) explored the work-related activities of female married distance education students, (2) identified school-related perceived stressors of female married distance education students and (3) examined the intentions of female married distance education students to quit their programme. Investigating the role of educational stress on intentions to quit or drop out is especially relevant as dropout intentions may be seen as a coping mechanism in response to stress (escape-avoidance coping) [67].

## Methods and materials

### Study design

The research adopted the sequential explanatory mixed-methods design, which started with a quantitative study followed by a purposeful qualitative phase based on the findings from the first stage. The use of this research design is justified because the investigators are interested in the attainment of both in-depth stress-related experiences and general realities that characterise the numerous loci of distance education students’ challenges in Ghana that research to date has marginalised [68–70]. Given the sparse research on this research theme across the sample, using the sequential explanatory mixed-methods approach is recognised as the most preferred design for this detailed inquiry. The subsequent sections of the methods have been structured into the quantitative and qualitative phases. The point of integration for this research is at the study design level (via explanatory sequential), method level (via the building approach) and interpretation stage (through the contiguous integration method).

### Quantitative phase

#### Participants’ information

The study sampled 164 female married distance education students from the University of Cape Coast (UCC), Cape Coast, Ghana. In terms of age, the youngest respondent was 23 years, whereas the oldest was 53 years old. The mean age was 35 years (*SD =* 6.08), indicating that the majority of the participants were in their middle adulthood stage. The participants’ reported number of children were 0 (*n =* 28, 17.1%), 1 (*n =* 30, 18.3%), 2 (*n =* 52, 31.7%), 3(*n =* 37, 22.6%), and 4 (*n =* 17, 10.4%). The mean number of children was 2 with a standard deviation of 0.23. Over 90% of the respondents were Christians (*n =* 150, 91.5%), with 7.3% being Muslims (*n =* 12) and 1.2% being atheists (*n =* 2).

#### Inclusion criteria

Due to the nature of the study population, the following inclusion criteria were used: (1) the student should be a registered postgraduate student in the selected institution, (2) the participant should be reading a postgraduate programme with both taught courses and research work (i.e., project work, dissertation or thesis), and (3) the participants must be of sound mind to give consent of participation.

#### Research instrument: Questionnaire

A questionnaire was designed and validated by the investigators for the quantitative phase of the research. The questionnaire had a number of items which were aligned with the research objectives. First, the demographic section was created, which had three items on the participants’ background information, namely, age, religion and the number of children. Other items were designed to obtain the work-related schedules of the respondents. Sample items include: What is your employment status (full-time vs. part-time vs. unemployed)? How many days do you spend at work, if you are working? How many hours do you spend at work, if you are working? Another section of the questionnaire asked participants to indicate, from a list of school-related stressors, the ones which place much burden on them. The instruction was “Rate the following school activity/activities on the extent to which they place much burden on you?” using a scale of 0 to 5, with 0 depicting no burden and 5 signifying huge burden. The options included attending lectures, engaging in individual or group presentations, presenting/submitting term papers, writing quizzes and exams and carrying out mandatory research work (e.g., dissertation, thesis). The last question was, “How often do you feel stressed by the activities of this programme?”. The items were developed based on extensive literature review related to the field of stress and distance education [49, 50, 54, 57, 62]. The questionnaire was developed adhering to the BRUSO approach (Brief, Relevant, Unambiguous, Specific and Objective) as have been adopted and utilised in previous studies [71]. Further, the content validity of the questionnaire was also established by experts in the fields of research methodology, clinical and health psychology, distance education, and quantitative psychology. The review comments, suggestions and recommendations were incorporated to improve the quality of the instrument [72]. As a preliminary check, five female married distance education students were identified and the questionnaire was administered to them. After the administration, their inputs concerning the clarity and understanding of the items were provided, which further shaped the items and the questionnaire.

#### Data collection procedures

All participants who identified themselves as satisfying all the inclusion criteria were contacted to participate in the study. Whereas some participants opted for an online version of the questionnaire (which was sent to their emails using Google forms), others preferred hard copies which were administered to them in class. The participants were assured of confidentiality, anonymity and volition, and consent was obtained through signing a consent form. After the questionnaire administration, the participants were asked to optionally indicate whether they would be willing to participate in the second phase (i.e., qualitative component) of the research. Those who gave such consent were required to provide other personal contact details on their questionnaire. About 44.5% (*n =* 73) of them indicated their willingness to participate in the qualitative phase of the research. Ethical approval was sought from the Institutional Review Board of the University of Cape Coast, Ghana, with reference number UCCIRB/EXT/2020/25.

#### Quantitative data analysis

The data retrieved from the participants were screened for data entry errors. All inconsistent data were removed from the dataset. The quantitative data were analysed using descriptive statistics, including frequency, percentage, mean, standard deviation, and correlational analysis. Bar and pie graphs were used to represent some portions of the data to address the objectives. More precisely, the frequency counts and percentage of the responses were computed on a number of work-related activities (e.g., number of days, how many hours of work, working full time, part-time or unemployed) in order to address research objective one. For research objective two, responses on the various stressors encountered by the female distance education students were summarised using mean and standard deviation. Whereas a correlational analysis was perfomed to examine the association among the identified stressors, frequency counts were used to assess the degree of stress they encountered; these are additional analyses to address the second objective. Data on the last research objective was analysed using frequency and percentage counts to tally the responses provided on the students’ intentions to quit their programme.

### Qualitative phase

The qualitative phase was drawn from the face-to-face interview and phone interview data collected from participants who opted to be part of the second phase of the research. This follow-up interview was necessary based on the following objectives: (1) to explain why most participants experienced stress, and (2) reasons for their intentions to quit the postgraduate programme, as shown in the first phase of the study.

#### Participants’ selection

The study purposefully selected individuals who opted to be part of the second phase. Although 73 participants opted to be part, we could not reach 30 of the participants through the contact information they provided. Out of the remaining 43, 11 of them had earlier indicated that they had not been stressed and had never thought of quitting the programme. Based on data saturation, we interviewed 10 out of the 32 participants who were available and willing to be interviewed face-to-face or via phone due to distance. It must be noted that all 32 had mentioned in the quantitative phase that they were experiencing stress and as well had intentions of quitting the programme.

#### Research instrument: Interview guide

A semi-structured interview guide was used to gather data from participants during the second phase of the study. Per the nature of the research design, the interview guide was carefully developed by the investigators based on the outcome of the quantitative phase of the research. The instrument focused on understanding participants’ stress and thoughts of quitting the programme as a follow-up on the initial survey. The interview questions bothered on getting in-depth information about the stress and stressors encountered in their schooling. The interview guide was first independently validated by experts in the fields of research methodology, clinical and health psychology, distance education, and educational psychology. Secondly, these experts together with the investigators had extensive discussion on the developed interview guide, afterwhich modifications were made accordingly.

Before the interview, participants were contacted and informed about the interviews. The participants and the researchers discussed and scheduled the date and time for the interviews, prioritising participants’ convenience to ensure their maximum cooperation and participation. Due to the difficulties of physical location of the homes of the study participants most (*n =* 7) of the participants were interviewed via phone based on preference, while the remaining (*n =* 3) were interviewed face-to-face. Participants consented to be interviewed and audiotaped by signing a consent form sent via WhatsApp and email to participants who were interviewed via phone. A hard copy of the consent form was made available for participants who were interviewed face-to-face to sign.

#### Qualitative data analysis

Transcripts were coded and thematically analysed using both manual and computer-assisted qualitative data analysis. To address the qualitative research objectives, a thematic analysis was carried out using the MAXQDA qualitative software for coding and development of themes. The qualitative data collected was first transcribed verbatim [73]. We read through the entire data to familiarise ourselves with the data, after which important sections were coded [74]. Initially, 84 codes were developed. These codes were then organised into two major themes [74]. The first major theme had five sub-themes, while the second major theme has two sub-themes (See Table 1). To ensure the trustworthiness of the qualitative results, the results were sent to participants to check the accuracy of the results regarding their experiences [75, 76]. Participants’ feedback did not have any significant influence on our findings. Students experiencing stress, as indicated in the quantitative phase, were involved in the qualitative phase to ensure the transferability of the findings to a similar population. Pseudonyms were also used to anonymise the identity of the participants.

**Table 1:** Major and sub-themes

Major themes	Sub-themes
Institutional/administrative stressors	Inconsistent schedules
	Unresolved complaints
	Academic workload
	Financial strain
	Discouragement
Personal stressors	Work/Employment
	Family demands

## Quantitative results

### Work-related activities of female married distance education students

Three indicators were examined to understand the work activities of the participants. These indicators were the employment status of the participants, the number of days of working (if they are employed) and the number of hours they worked. The details of the analysis are shown in Table 2.

**Table 2:** Work schedules of female married DE students

Variables	Response	Frequency	Percent
Employment status	Full-time	141	86.0
	Part-time	16	9.8
	Unemployed	7	4.3
Number of days for work in a week	0 day*	7	4.3
	1 day	0	0
	2 days	0	0
	3 days	0	0
	4 days	0	0
	5 days	128	78.0
	6 days	14	8.5
	7 days	15	9.1
Number of hours of work in a day	0 h*	7	4.3
	5 h	2	1.2
	6 h	2	1.2
	7 h	8	4.9
	8 h	80	48.8
	9 h	25	15.2
	10 h	25	15.2
	11 h	3	1.8
	12 h	12	7.3

* These respondents were not employed

A greater percentage of the participants were employed in full-time jobs (86%), with less than 10% working as part-time workers (9.8%) (see Table 2). Very few participants were unemployed (*n* = 7, 4.3%). The majority of the students worked 5 days a week (*n* = 128, 78%). Others reported working 6 days (*n* = 14, 8.5%) and 7 days (*n* = 15, 9.1%) within the week. Concerning the number of hours, it was revealed that most of the participants worked 8 h or more within the day. For example, 48.8% worked for 8 h, 15.2% worked for 9 h and 15.2% again worked for 10 h. Some also reported working for 11 h (1.8%) and 12 h (7.3%) in a day.

### School-related perceived stressors on female married distance education students

The study examined 5 main school-related perceived stressors of postgraduate studies and attempted to understand which of them placed much stress on them. The study also explored how burdens on a particular school activity could spill over to other related activities. The results are shown in Table 3.

**Table 3:** Ratings of school activities based on the associated burdens

SN	School Activities	Mean	SD	1	2	3	4
1	Attending lectures	2.48	1.50	1			
2	Engaging in presentation	2.31	1.28	0.397^**^	1		
3	Writing term papers	2.38	1.30	0.289^**^	0.529^**^	1	
4	Writing exams	2.74	1.51	0.395^**^	0.271^**^	0.291^**^	1
5	Conducting research	3.49	1.41	0.173^*^	0.355^**^	0.424^**^	0.366^**^

**. Correlation is significant at the 0.01 level (2-tailed)

*. Correlation is significant at the 0.05 level (2-tailed)

As shown in Table 3, it was discovered that conducting research work placed the highest stress on the students (*M* = 3.49, *SD* = 1.41), followed by writing examinations (*M* = 2.74, *SD* = 1.51) and attending lectures (*M* = 2.48, *SD =* 1.50). Producing term papers and doing individual or group presentations in class were considered the least in terms of stress. Further results also showed significant positive relationships among the school activities regarding the associated stressful ratings. In particular, the relationship coefficients ranged from 0.173 to 0.529, suggesting that when there is a higher stress level associated with any of the activities, it is likely that other activities would also place a huge burden on the students.

**Fig. 1:**
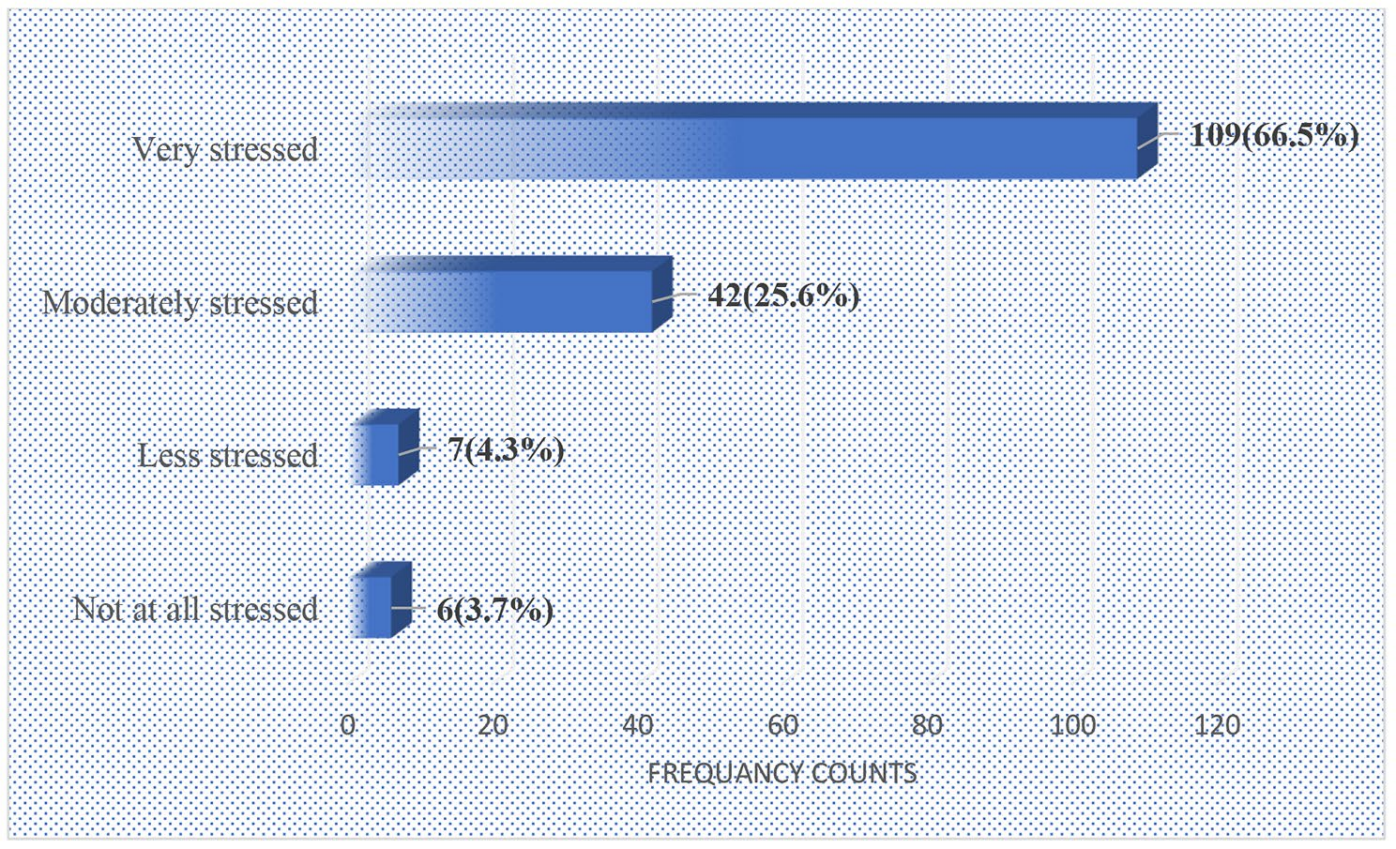
Degree of stress encountered on the postgraduate programme

The respondents were also asked to indicate how stressful they felt being on the programme. As depicted in Fig. 1, it was revealed that about two-thirds of the female married distance education students felt much stressed (66.5%). Other participants also reported being moderately stressed (25.6%) and only 4.3% of them indicated less stressed. About 3.7% of the participants mentioned that they were not stressed at all.

### Intentions of female married DE students to quit their programme

**Fig. 2:**
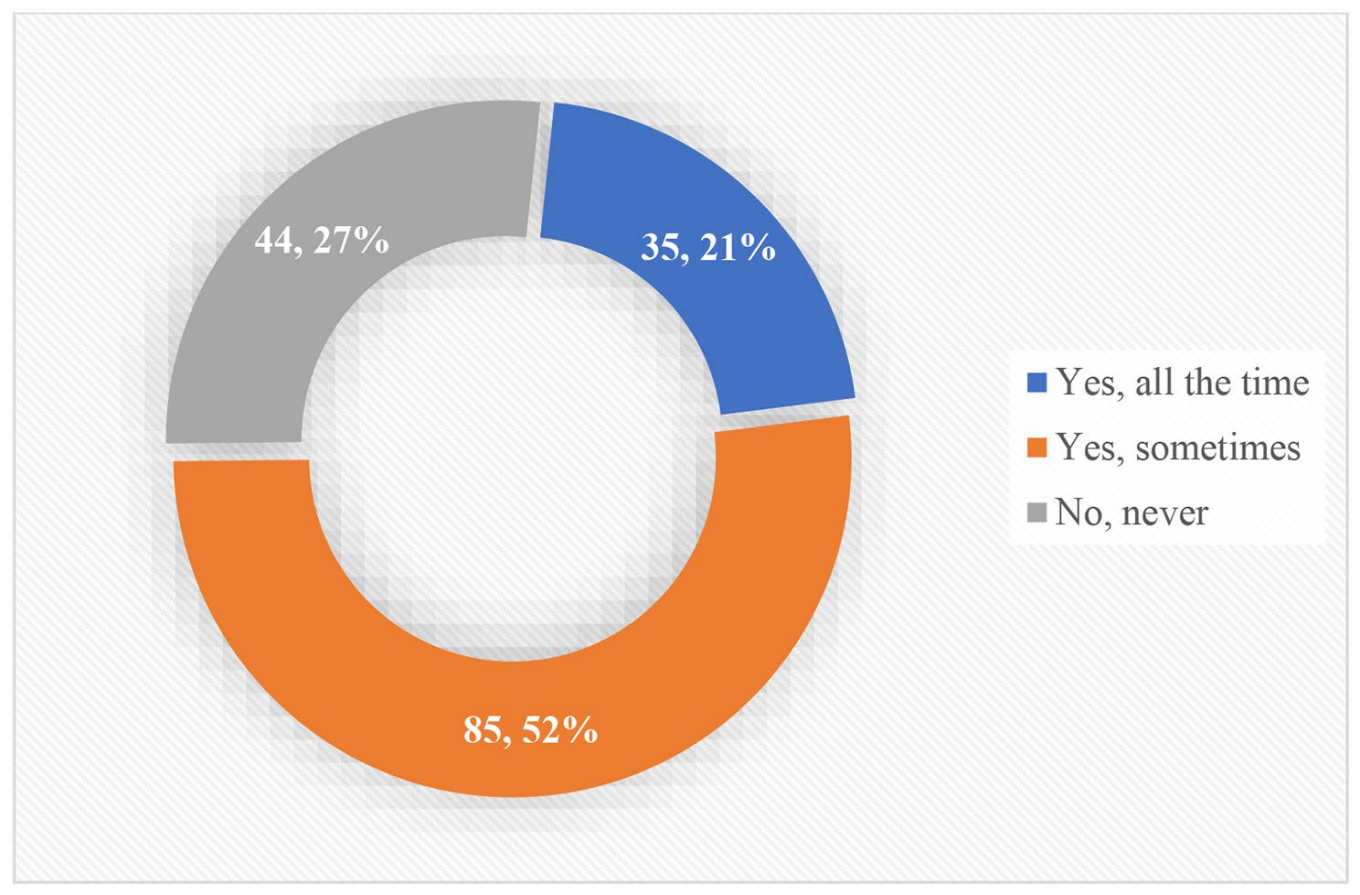
Intentions to quit postgraduate studies

The study also sought to assess respondents’ intentions to terminate their postgraduate studies due to its stressful nature and other associated factors. The graphical analysis as depicted in Fig. 2 showed that about 21% of the students had intentions to quit the programme, at all time (*n =* 35). Furthermore, a larger proportion of the students reiterated that they sometimes have thoughts of quitting their postgraduate studies (*n =* 85, 52%). However, some students reported that they had never thought of quitting the programme (*n =* 44, 27%).

### Qualitative results

#### Participants’ views and explanation of themes

Distance education allows students and teachers flexibility regarding place, time and pace of learning [54, 77]. Distance education students/learners choose not to attend the traditional classroom or face-to-face schooling for several reasons, such as time constraints, geography, family demands and work requirements [78]. In turn, students have the advantage of deciding their learning. This suggests that distance education’s flexibility will reduce stress (distress). However, this was not the case for the study participants. Instead, the themes that emerged from the data show that most participants experienced stress. Hence, participants thought of quitting the programme. Two major themes and five sub-themes were generated after coding and categorisation of the codes. These themes and sub-themes are presented in Table 1.

#### Institutional/administrative stressors

Inconsistent schedules, academic workload, unresolved complaints, financial strain and discouragement, were the primary sources of the institutional/administrative stressors which made the study participants think of quitting the programme. In addition, participants spoke about how they are stressed due to the administrative activities of the distance education programme.

#### Inconsistent schedules

Participants explained that their thoughts of quitting the programme emanate from the stress they experience due to inconsistent schedules and postponement of activities such as the distribution of course materials, quizzes and examinations. Some participants said:

*“…it is much stress when you prepare for an examination and just a day before the examination it is postponed…”* (Keziah).

*“…they [*referring to the University management*] are not consistent with the timetable they give us; they keep changing and that stresses me because it makes me keep changing my schedules at work …”* (Hannah).

Another participant illustrated how students are stressed:

*“The organisation of the school and the way of distributing modules [referring to reading materials] is appalling because it prevents us from studying since we don’t have the modules only to receive a module a week before the exam”.* (Anita)

### Unresolved complaints

Consumers expect that when they express their dissatisfaction about products, services or staffs of an organisation or institution, it would be acknowledged and the necessary actions carried out to ensure their satisfaction or grievances addressed. This makes the organisation or institution accountable to the public, improving its reputation and strengthening public confidence [79]. However, the findings of this study show that students’ complaints were unresolved. The participants explained that the unresolved complaint stresses them, so they thought of quitting the programme. Some participants expressed:

*“Sometimes, if you have a problem, you complain several times, but it wouldn’t be solved until you go to the main campus.”* (Keziah).

Another participant added that

*“…also, some papers haven’t appeared on my results slip [transcript], and getting help to resolve it has taken me a year, but still it has not been resolved, and in fact, it frustrates me…”* (Rebecca).

### Academic workload

Most of the participants in this study reported being stressed due to the academic workload. They explained that many topics are taught within a day, so they have to spend much time reading their course materials. In addition, the participants explained that they are also given several assignments to complete, which adds to their workload. The following are extracts from the participants’ interviews:

*“Yes, I once thought of stopping because of the workload. Sometimes, many different topics are taught on the same day, and I have three or more assignments to do at a time which I have little time for…”* (Hannah).

*“…I am thinking of stopping because the workload is too much for me to handle. We are given too many assignments.”* (Rebecca).

The findings of this study imply that the distance education students involved in this study need much time to meet their academic demands. Hence, if a student enrolled on a distance education programme because of time constrain, as it has been identified by some studies [7, 80] as one of the reasons why some individuals opt for distance education/learning, then it is likely that those individuals might put their thought of quitting the programme into action.

### Financial strain

Financial strain was an issue of concern to some participants. The participants explained that stress resulting from financial difficulties fuels their intentions to quit the programme. They complained of expensive tuition fees and limited time of payment. According to some participants:

*“Another reason is that the school fees [tuition fees] is too much [expensive] as compared to other institutions that offer a similar programme.”* (Bridgit).

*“Also, the amount of fees is expensive and the duration to make payment before our courses are registered is too short for raising that amount to pay the fees”* (Leticia).

### Discouragement

Many students hope to secure better employment or be promoted in their careers or work after graduation. In this study, most of the participants are workers seeking to upgrade themselves or enhancing their professional development but have to opt for distance education due to work schedules. However, a participant shared her concerns about how some teachers discourage them. She explained that some lecturers retort that they would not be upgraded because her degree was obtained through distance education. She said:

*“You know, most of us are teachers and want to upgrade, but it’s too stressful and it hurt to hear some of our teachers saying that after the distance programme, it won’t send you anywhere using your certificate…”* (Anna).

### Personal stressors

An advantage of distance education/learning is the flexibility of time and the opportunity for students to plan their learning and complete their academic courses at their own pace [81]. Therefore individuals who are not able to attend traditional face-to-face school due to but not limited to, work, financial, geographic, family and time constraints usually opt for distance education so that they can achieve their academic goals without interfering with or disrupting their personal everyday life [81]. However, our analysis revealed that work/employment and family demands (sub-themes) were the personal stressors that made participants consider quitting the programme.

### Work/employment

Strain resulting from work/employment was a significant personal stressor, according to most of the participants. During our interview, most participants explained that they had thought of quitting their programme because combining work and academics was challenging since both require time and attention. They added that sometimes they have to be at work, and at the same time, they have to be at lectures. A participant illustrated how the combination of her work and academic work stresses her:

“*I am a worker; throughout the week, I am at work and leave (close from work) around 6 pm or even beyond. Therefore, sometimes it is not easy to get time to learn. Also, sometimes deadlines given for the submission of assignments are very short, putting us under a lot of pressure and stress. For that reason, it occurred to me once to quit. But I still gathered the strength to go on.* (Leticia)

Some participants added:

*“It is stressful combining examinations, projects, presentations, assignments, quizzes, managing my family, monetary issues, and online lectures…” Sometimes I feel I should just give up.”* (Dorine).

A participant also said:

*“I have no time at all; every day, I am busy. I go to work from Monday to Friday and sometimes close very late and tired, so I am not able to study…”* (Anna).

### Family demands

Participants explained that combining family demands such as cooking and taking care of children with academic work such as completing assignments and attending lectures is strenuous, so sometimes, they are tempted to quit studies. A participant illustrated how the need to meet her family demands and academic work stresses her:

*“…also, I have to take care of the family and make sure our home is in order. So, it is very stressful to do all of these and assignments, prepare for quizzes and examinations.”* (Anna).

Another participant said:

*“I have thoughts of stopping because I hardly get someone to take care of my kids on Saturdays as my husband, too, is a health professional who goes to work on Saturdays. Travelling from my place of residence to Ho [the capital of the Volta Region where the study centre is located] along with my little children, is stressful”* (Gloria).

Another participant, who looked frustrated, said

*“Sometimes, I want to quit because of family pressure, especially when I am learning.*” (Bridgit).

## Discussion

The current inquiry investigated the work-related activities of female married distance education students, identify school-related perceived stressors of female married distance education students and examine their intentions to quit their programme. The findings indicate that the majority of the female married postgraduate students were full-time workers, with many working five days a week and eight hours a day. Additionally, the study findings showed that most of the students reported that conducting research imposes a higher burden of stress with the least reported stressors been term papers and individual/group presentations. Further, the research findings revealed that the majority of the students sometimes thought of quitting their postgraduate studies with few indicating they never thought of quitting the programme. The findings in this study are novel as it provides a qualitative perspective to help deepen the understanding of stress-related experiences of postgraduate students in the distance education programme. The general implication of these findings is that participants who enrolled on the distance education programme enjoy the benefit it offers about time flexibility [54, 78, 80], however, in meeting family demands, they become disappointed at a point of their studies. This latter outcome is a result of the possible effect of stress arising from the various demands (i.e., academic, work and family management) that impose a psychological burden that affects one’s quality of life. This result may therefore lead to attrition from the programme, poor academic performance, suicidal ideation, role conflict, decreased intrinsic motivation and many more negative tendencies. For instance, the findings showed that the majority of the students were in full-time employment, working five days a week and eight hours a day. This work-related engagement, when combined with academic demands, more so at the postgraduate level, may manifest negative physical, psychological and academic impacts [59, 82]. Though related studies are rare to contrast, one analogous study found increased working hours and days to be associated with stress [83], which was also confirmed in the qualitative finding. Moreover, most female postgraduate distance education students have family-related responsibilities, such as nursing babies, which further compound their academic challenges. Plausibly, this observation could be the reason majority of the participants reported their intentions of quitting the programme. This argument is buttressed by qualitative findings, where the participants reiterated the stressful demand of caring for the family as an impediment to their academic progression. Alabi et al. [83] argued that the socio-cultural discrimination against women in Africa challenges their higher academic achievements. Since married women are entirely saddled with family care responsibilities which have a competing interest against their higher academic demands, therefore, these demands compel most women not to pursue higher education. These women opt for programmes like distance education just to be available at home to take care of the family, which comes with stressful demands [84].

Invariably, the findings revealed that most participants sometimes have thoughts of quitting their postgraduate education, with few indicating never thought of quitting the programme. This outcome means that the students may have reached a mental state of distress due to dissipated intrinsic and extrinsic motivated factors and poor system support either from the school, workplace and family support systems. By providing interventions that have proved to reduce stress and improve coping mechanisms and stress management, educational institutions can attempt to reduce the academic-related stress experienced by students. Also, extant literature has shown that educational programmes that increase students’ stress-coping abilities and skills directly and favourably affect academic achievement and reduce health risks [85–87]. For instance, a meta-analysis of 19 randomised controlled trials or quasi-experimental research discovered that student coping abilities were enhanced and stress symptoms were reduced by school programmes aimed at stress management [88]. A similar study in South Africa upheld the findings of the present study. For example, Silinda and Brubacher [89] found that postgraduate students in distance education programmes considered quitting the programme due to the accompanying overbearing stress and lack of support to mitigate challenges. The similarity in the finding may be due to the cross-cultural nature of academic-related stress issues. That notwithstanding, few students in the present study indicated no intention to quit the programme, which is a positive sign of resilience and noteworthy for further investigation in future research to find out their motivation. This outcome supports the assertion of Beccaria et al. [90] that these few students may be actively engaging in protective or resilience strategies to cope with their stressors, a situation worthy of emulation by others. Therefore, academic institutions running distance education programmes are encouraged to design and implement policies as well as active coping interventions that are learner-specific since individual students have different skills and motivation levels.

In furtherance, the findings also showed that most students reported that conducting research imposes a higher burden of stress with the least reported stressors being term papers and individual/group presentations. This result concurs with Silinda et al.’s [91] findings, where distance education students reported that dissertation and thesis writing imposes higher stress on them. The qualitative findings of Silinda and co’s study identified uncertainty about the research/writing process along with insufficient support from supervisors as their stressors and the reason for their intention to quit the programme. This link was confirmed in the qualitative aspect of the present study, where participants intimated institutional stressors such as inconsistent schedules, academic workload, unresolved complaints, and financial strain as precursors for their stress and intentions to abrogate their various programmes. These findings revealed the lack of or inadequate student support systems within the distance education programme of postgraduate education in Ghana. Besides, even if these support systems are available, they may not be effective in ameliorating the academic stress of students and need to be reviewed. This situation has been advocated for by the international “Healthy Universities” movement, which promotes the university’s role as a resource for promoting health and well-being among students, faculty, and the general society through instruction, research, information exchange, and institutional practice and not just strictly academics to the neglect of the students’ well-being [89].

The findings imply that institutions offering postgraduate distance learning programmes in Ghana should aid their students by providing training or counselling services that would cater for the needs of the distance learners, such as how to balance academic work with family and employment duties through integrated planning. Other implications include enhancing supervisors’ support of distance learning postgraduate students, such as through better communication, faster feedback delivery, and clearer correspondence, to help ensure that these students receive the guidance they need when writing their dissertation or thesis.

### Strengths and limitations

The study’s relevance and strengths are bolstered using a two-prong mixed-methods perspective of evidence gathering to deepen and broaden understanding of the stress-related phenomena. Despite the study’s advantages, a few restrictions must be highlighted. First, it is structured as a cross-sectional design that examines the real world at a certain period. Such a strategy does not investigate longitudinal variations in perceived stressors across time. Also, we cannot completely rule out information bias because the data were gathered via self-administered questionnaires and/or devices. However, non of these limitations invalidate the findings of the study.

### Conclusion and recommendations

This study investigated work-related activities of female married distance education students, identified school-related perceived stressors of female married distance education students and examined the intentions of female married distance education students to quit their programme. It can be concluded from this study that female postgraduate distance education students perform multiple roles as full-time employees with family and academic demands that can have a negative health and academic impact. Also, the majority of female postgraduate distance education students have a high propensity to quit their studies due to a lack of support from both academics and family. Lastly, conducting research imposes a high burden of stress on the participants compared to other academic-related activities.

Therefore, postgraduate distance learning students are encouraged to focus more on using active or functional coping strategies (e.g., integrated planning) rather than adopting behavioural disengagement. Active coping will assist distance learners to better initiate forearmed preparations for similar situations in the future and acquire relevant abilities to manage potential stressors. Also, orientation programmes on active coping methods offered by educational institutions and employers will be highly beneficial in helping distant learners achieve this aim. It is also advised that educational institutions provide academic counsellors to distant learners. These counsellors will advise distant learners on how to handle or manage their difficulties while pursuing their academic goals.

## Data Availability

Anonymized data is available upon reasonable request through the corresponding author.

## Declarations

### Competing of interests

None declared.
